# A prospective cohort study of prognosis for newly diagnosed epilepsy in east China

**DOI:** 10.1186/1471-2377-13-116

**Published:** 2013-09-04

**Authors:** Yanfang Zhang, Nian Yu, Lingying Su, Qing Di

**Affiliations:** 1 Department of Neurology, Nanjing Brain Hospital affiliated to Nanjing Medical University, 264 Guangzhou Road, Nanjing, Jiangsu Province, China

**Keywords:** Antiepileptic drugs, Clinical pattern, Drug-resistant epilepsy, Prognosis, Risk factors

## Abstract

**Background:**

Limited data are available on the outcome of antiepileptic drug treatment response in patients of Chinese Han ethnicity with newly diagnosed epilepsy. We sought to explore the prognosis with antiepileptic drug treatment and to identify the predictors of poor drug control of seizures in these patients.

**Methods:**

For at least 2 years, we prospectively followed up a cohort of patients with newly diagnosed epilepsy and analyzed the response to each antiepileptic drug. Cumulative risk for seizure relapse after initial remission achieved was estimated. The patients were divided into two groups (poor and good control) and compared for clinical characteristics.

**Results:**

A total of 180 patients were included. Early remission was reached in 125 (69.44%) patients, 19 (10.56%) patients entered late remission, while 36 (20%) patients failed to achieve remission. The relapse rates were 19.5% at 2 years and 31.9% at 3 years of the follow-up. The response rates of the first throughout the fourth treatment regimens were 60.0%, 16.1%, 2.8%, and 0.6%, respectively. Multiple seizure types and changes in seizure type during treatment were significantly (*p* = 0.013 and 0.047, respectively) associated with a poor control.

**Conclusions:**

The prognosis of the majority of patients with newly diagnosed epilepsy is good and the clinical pattern of epilepsy during treatment is complex. The chances of seizure control declines with each subsequent treatment regimen. The prognosis for patients with multiple seizure types and seizure type changes during treatment is unfavorable.

## Background

Epilepsy is one of the most common neurological conditions. Seventy million people have epilepsy, with the incidence of 34 – 76 of newly diagnosed cases per 100,000 [1]. While the majority of patients with epilepsy respond well to one antiepileptic drug (AED), nearly up to one third of patients respond poorly to antiepileptic therapy with two or more AEDs, or develop drug-resistant epilepsy (DRE). Uncontrolled epilepsy and overdose of AEDs are associated with adverse effects, such as cognitive deterioration, psychosocial dysfunction, and increased morbidity and mortality [2,3]. Therefore, early identification of patients who are at high risk of developing DRE is crucial.

While the risk factors influencing the prognosis of epilepsy have begun to be appreciated [4-7], limited data are available on the clinical patterns of treatment response in newly diagnosed epilepsy. It is important to understand the different clinical patterns of response to AED treatment, ideally by following the outcomes once the treatment has been initiated. However, some current studies were limited by selection bias toward patients with drug-resistant epilepsy who had already failed at least two drug regimens [8,9]. Further, epilepsy has heterogeneous etiology and outcomes. The disease patterns in individual patients may be genetically determined and, thus, may vary among patients from different ethnic backgrounds. Currently, a large number of epileptic individuals (estimated over six million sufferers) live in China [10], but there is no data in this population, necessitating studies on this field.

In the present study, we prospectively followed up a cohort of patients in east China with newly diagnosed epilepsy to explore the prognosis with antiepileptic drug treatment and to identify the predictors of poor drug control of seizures in these patients.

## Methods

### Patients

In 2005, the International League against Epilepsy (ILAE) re-defined epilepsy as a disorder of the brain characterized by an enduring predisposition to generate epileptic seizures and by the neurobiologic, cognitive, psychological, and social consequences of this condition. The definition of epilepsy requires the occurrence of at least one epileptic seizure [11]. In our study, patients who had never previously received treatment with AED and who met the new definition of epilepsy were recruited from the Neurology Clinic of Nanjing Brain Hospital between January 2000 and June 2010. The patients were prospectively followed up until the end of June 2012, i.e., for at least two years. Informed consent was obtained from each participant. The study was approved by the Nanjing Brain Hospital affiliated to Nanjing Medical University Ethics Committee.

During the first visit, we collected demographic and clinical information from patients and their relatives using a structured questionnaire developed in-house, and performed general physical and neurologic examinations. Electroencephalography (EEG) was performed in patients to facilitate classification of the epilepsy, including video EEG monitoring within 24 hours and standard stimulation procedures (photic stimulation and hyperventilation). Magnetic resonance imaging (MRI) of the brain was performed using a high resolution 3.0 T. All MRI scans were conducted by a specialized neuroradiologist using standard MR protocols to screen for underlying structural abnormalities that might have caused the epilepsy. Patients with serious systemic illnesses or seizures provoked by external factors, e.g., alcohol withdrawal, were excluded at the time of analysis.

Possible determinants were obtained prior to diagnosis and initiation of the treatment and included patients’ gender, age at the seizure onset, duration of epilepsy, seizure frequency, presence of seizures during sleep, seizure type, changes in seizure type during treatment, etiology (i.e., genetic, structural/metabolic such as head trauma, tumor, stroke, infection), family history of epilepsy, febrile seizures, history of brain injury, early mental retardation, and brain MRI and EEG findings. For either generalized onset epilepsies or focal onset epilepsies, seizures would be presented in different forms in clinical phenomenology. Multiple seizure types may coexist and the type may change during different periods in some epilepsy syndromes. So, the two factors were used to predict the outcome of epilepsy in our study. Duration of epilepsy was defined as the period from the seizure onset to the end of follow-up. Seizure frequency was defined as the mean monthly seizure frequency within 1 year before treatment. A positive family history was defined as the presence of epilepsy in first-degree relatives (i.e., parents, siblings, and children).

### Definitions

According to the ILAE classification of epileptic seizures [12], seizure types were categorized into generalized (tonic, clonic, or tonic–clonic) or focal (simple or complex partial). The epilepsy syndrome was classified as genetic, structural/metabolic, unknown cause, using the ILAE classification criteria of epilepsies and epileptic syndromes [13].

Outcomes were obtained from personal interviews. Remission was defined as an achievement of at least one year free of seizures, and categorized into early and late remissions. Early remission was achieved within first year of treatment initiation, as opposed to late remission, which was achieved after more than first year of treatment. Terminal remission was regarded as remission achieved at the end of follow-up. Relapse described the occurrence of repeated seizures after remission achieved. According to the definition proposed by the ILAE [14], DRE was defined as the failure of two well-tolerated, and appropriately chosen and used AED schedules, whether as monotherapies or in combination, to achieve a sustained seizure freedom for either one year or for a period equal to three times of the pre-intervention inter-seizure time, whichever was longer.

### Treatment

The treatment given in the present study was done as part of standard care. Patients were prescribed AEDs according to their seizure type, personal profiles (i.e., sex, age)and drug characteristics (i.e., efficacy, side effects and interaction profiles). The AEDs we have prescribed in the study include carbamazepine, valproate sodium, oxcarbazepine, lamotrigine and topiramate. Monotherapy was tried initially in all patients. AEDs were increased to the maximum tolerated doses. Patients who continued to experience seizures despite at high doses of AED were designated as treatment failures because of lack of efficacy. Those developing idiosyncratic reactions or experiencing intolerable side effects at low AED dosage were deemed to have failed treatment because of adverse effects. If the first prescribed AED is poorly tolerated at low dosage or fails to improve seizure control, patients would be prescribed another drug. If the first AED is well-tolerated but does not completely abolish the seizures, combination therapy would be applied. Compliance with the treatment regimen was monitored at the clinic. Patients who did not comply with the treatment regimen were excluded from the study.

### Follow-up and outcome

Patients were evaluated at 4 weeks after the treatment started and then at 3-month intervals thereafter. At each follow-up visit, seizure frequency, drug doses, response to drug therapy and compliance were routinely recorded and adjusted, as dictated by clinical circumstances. The follow-up data were collected on a data record sheet specially developed for the purpose of this study.

The final evaluation of seizure control was performed after a minimum of 2 years of follow-up. For comparison, we divided patients into two groups. Patients who met the definition of DRE were considered to have a poor prognosis. The remaining patients were labeled as having a good prognosis.

### Statistical analysis

All analyses were performed with SPSS 13.0 software (IBM, Chicago, USA). The two-tailed chi-square or Fisher’s exact tests were used for comparison of categorical data, while the Student’s *t* test or the Mann–Whitney test were used for comparison of continuous data. Among those patients who achieved one year seizure remission, Kaplan-Meier analyses were used to estimate the cumulative risk for the seizure relapse. Logistic regression was used to investigate covariates of interest, first individually in univariate models and then together in a multivariate model. The odds ratio (OR) and 95% confidence interval (CI) were calculated. The two-tailed *p* value of < 0.05 was considered as statistically significant.

## Results

### Patient characteristics

A total of 212 patients were diagnosed with epilepsy and none had previously received an AED for any indication. Thirty-two patients (15.1%) were excluded from analysis because of lack of sufficient follow-up information. At the end of the follow-up period, outcomes were known for the remaining 180 (84.9%) patients. EEG was performed for each patient and 35 of them accepted video EEG monitoring. The median duration of the follow-up was 5 years (range 2 – 10 years). Among the 180 patients included in the study, 94 (52.2%) were male. The median (range) age at referral was 19 (6 – 71) years, and the median (range) age at the onset of epilepsy was 13 (1 – 65) years. A slightly higher number of patients (56.1%) had focal seizures, while the remaining patients (43.9%) presented with generalized seizures. Epilepsy was classified as genetic in 47 (26.1%), structural/metabolic in 55 (30.6%), and unknown cause in 78 (43.3%) patients. Structural damage of the brain was caused by stroke, trauma, tumor, infection, tuberous sclerosis and cortical malformations. Metabolic etiologies mainly include mitochondrial disorders.

### Prognosis of patients with newly diagnosed epilepsy

**Figure 1:**
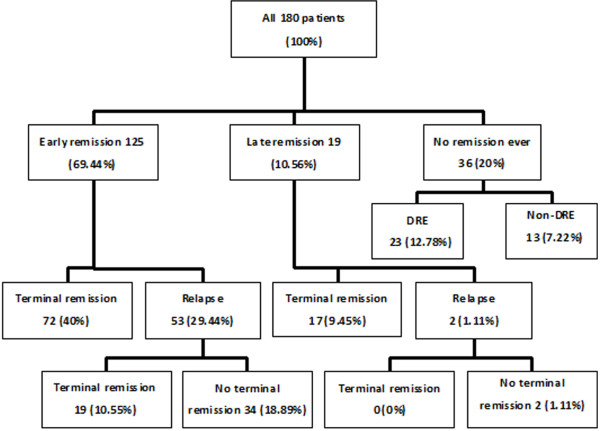
**Outcomes for patients with newly diagnosed epilepsy.** The treatment outcomes of patients were divided into three groups: early remission (seizure-free disease achieved for at least one year within the first year of starting the therapy), late remission (seizure-free disease achieved for at least one year after more than 1 year of therapy), and no remission ever (never achieved at least one year of seizure-free disease during the follow-up period).

In total, 144 (80%) patients remained seizure-free for at least one year. The remaining 36 (20%) patients never experienced a one year remission while continuing AED therapy (Figure 1).

#### Early remission

Early remission was reached in 125 (69.44%) out of 180 patients. Despite a good initial outcome, relapse occurred in 53 patients. The remaining 72 patients went into terminal remission with no relapse until the end of the follow-up period.

#### Late remission

Nineteen (10.56%) out of 180 patients entered late remission. One or more relapses were noted in 2 (1.11%) patients, but 17 (9.45%) patients remained in terminal remission without any relapse.

#### Remitting course of epilepsy

Terminal remission uninterrupted by relapse was noted in 72 (40%) patients in the early remission group and in 17 (9.45%) patients who entered the late remission phase. Overall, 89 out of 180 (49.44%) patients reached the terminal remission without relapse indicating a remitting course of epilepsy.

#### Remitting–relapsing course of epilepsy

**Figure 2:**
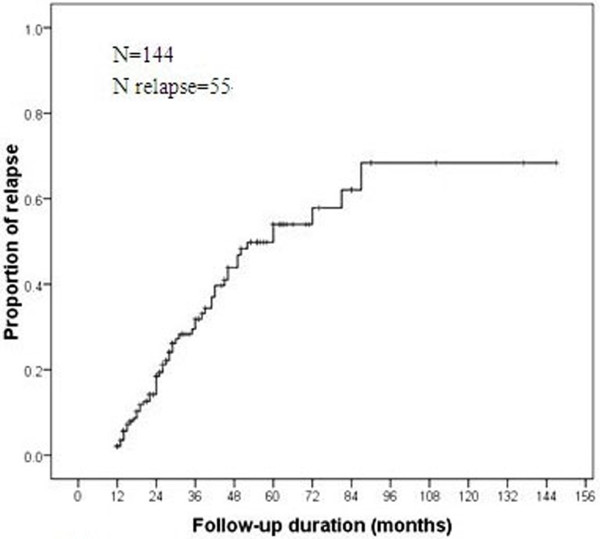
**The Kaplan–Meier estimate of cumulative probability for subsequent seizure relapse.** Initial remission was achieved in 144 patients, of whom 55 experience relapse. Kaplan–Meier estimates the cumulative probability of relapse during the follow-up period.

Out of 144 (30.55%) patients who achieved a one year remission, the events of relapse occurred in 55 patients, indicating a remitting–relapsing course of epilepsy. A Kaplan-Meier plot of the time to relapse showed a rate of 19.5% at 2 years and 31.9% at 3 years of follow-up (Figure 2).

#### Worsening course of epilepsy

Terminal remission after relapse was noted in 19 of 53 (10.55%) patients following early remission. None of the 2 patients, whose late remission was followed by relapse, regained terminal remission. There were 34 out of 125 (18.89%) patients, whose early remission was followed by relapse, and 2 out of 19 (1.11%) patients, whose late remission was followed by relapse; these 36 patients never regained terminal remission, resulting in a total of 20% (36/180) patients with a worsening course of epilepsy.

#### Drug resistance

In total, 36 (20%) patients never experienced a one year remission during the AED therapy. Among them, 23 (12.78%) patients failed to achieve remission after using at least two AEDs and were thus defined as having DRE. The remaining 13 (7.22%) patients with no remission only received one AED therapy and could not yet be classified as DRE.

### Efficacy of AED therapy

Among 180 patients with newly diagnosed epilepsy, 144 (80%) were seizure-free for at least one year of therapy. One-hundred eight (60%) patients achieved remission with the first AED, while 36 (20%) patients became seizure-free with subsequent drugs. The overall response rates for the first, second or third treatment schedules as proportions of the study population were 60.0%, 16.6% and 2.8%, respectively, with just one (0.6%) patient responding to further drug trials (Table 1).

**Table 1:** Relationship between the number of AEDs and the therapy outcome in the newly diagnosed epilepsy

**AED attempts**	**Number of patients (%)**	**Outcome**	
		**Good N (%)**	**Poor N (%)**
1	119 (66.1)	108 (90.8)	11 (9.2)
2	40 (22.2)	30 (75.0)	10 (25.0)
3	14 (7.8)	5 (35.7)	9 (64.3)
4	5 (2.8)	1 (20.0)	4 (80.0)
5	2 (1.1)	0 (0)	2 (100)
Total	180 (100)	144 (80.0)	36 (20.0)

AED: antiepileptic drug. A good outcome was defined as a seizure-free remission for at least one year. A poor outcome was defined as no seizure-free remission for one year. Percentages of the therapy outcome sum up by row.

In the remaining 72 patients who had uncontrolled seizures, 34 (47%) patients discontinued their first drug because of lack of efficacy, 30 (42%) because of intolerable adverse effects, 8 (11%) for other reasons, such as planning a pregnancy or a change of mind about drug treatment.

### Analysis of predictors related to seizure outcome

At the end of follow-up, 23 of the 36 (12.78%) patients who never experienced remission were defined as DRE and classified into the poor outcome group; the remaining 157 (87.22%) patients were classified into the good outcome group. The patients of these two groups did not differ significantly with regards to gender distribution, age, age at the seizure onset, and etiology (Table 2).

**Table 2:** Clinical characteristics of patients with the newly diagnosed epilepsy

**Disease parameter**	**Outcome**		***P *value**
	**Poor (N = 23)**	**Good (N = 157)**	
Male gender	11 (47.8%)	83 (52.9%)	0.651
Age	17 (9 – 37)	19 (6 – 71)	0.516
Age at the seizure onset	12 (4 – 30)	13 (1 – 65)	0.822
Duration of epilepsy (months)	81 (24 – 99)	40 (24 – 110)	**< 0.001**
Type of epilepsy			**0.022**
Focal seizures	18 (78.3%)	83 (52.9%)	
Generalized seizures	5 (21.7%)	74 (47.1%)	
Etiology			0.128
Genetic	9 (39.1%)	38 (24.2%)	
Structural/metabolic or unknown cause	14 (60.9%)	119 (75.8%)	

The bold in the table indicates statistically significant data.

A good outcome was defined as a seizure-free remission for at least one year. A poor outcome was defined as no seizure-free remission for one year. The percentages sum up by column.

By contrast, the duration of epilepsy was longer in the poor outcome group compared with the good outcome group (*p* < 0.001; Table 2). Further, a significantly higher proportion of patients had focal seizures in the poor outcome group (*p* = 0.022; Table 2).

Univariate logistic regression analysis of the patients in these two groups demonstrated that poor outcome was associated with focal seizures, multiple seizure types and changes in seizure type during therapy (Table 3). The multivariable logistic regression model further demonstrated that multiple seizure types (OR=3.33, 95% CI 1.29-8.60, *p* = 0.013) and changes in seizure type during treatment (OR=5.88, 95% CI 1.03-33.62, *p* = 0.047) were predictive of poor outcome (Table 4).

**Table 3:** Univariate comparison of outcomes in the newly diagnosed epilepsy

**Parameter**	**Outcome**		**Univariate comparison**		
	**Poor (N = 23)**	**Good (N= 157)**	**OR**	**95% CI**	***P *value**
Male gender	11 (47.8%)	83 (52.9%)	0.817	0.34 – 1.963	0.652
Age at the seizure onset	12 (4 – 30)	13 (1 – 65)	1.012	0.967 – 1.059	0.598
>1 seizure monthly before treatment	4 (17.4%)	26 (16.6%)	1.061	0.333 – 3.375	0.920
≥50% seizures during sleep	12 (52.5%)	81 (51.6%)	1.024	0.426 – 2.458	0.958
Focal seizures	18 (78.3%)	83 (52.9%)	3.21	1.135 – 9.073	**0.028**
Multiple seizure types	10 (43.5%)	27 (17.2%)	3.704	1.472 – 9.319	**0.005**
Changes in seizure type during treatment	3 (13.0%)	3 (1.9%)	7.70	1.454 – 40.77	**0.016**
Genetic epilepsy	9 (39.1%)	38 (24.2%)	2.013	0.807 – 5.019	0.133
Positive family history	2 (8.7%)	14 (8.9%)	0.973	0.206 – 4.587	0.972
Abnormal perinatal history	3 (13.0%)	11 (7.0%)	1.991	0.511 – 7.753	0.321
History of brain injury	1 (4.3%)	9 (5.7%)	0.747	0.09 – 6.19	0.787
Febrile seizures	4 (17.4%)	24 (15.3%)	1.167	0.365 – 3.731	0.795
Mental retardation	1 (4.3%)	4 (2.5%)	1.739	0.186 – 16.27	0.628
Abnormal neuroimaging	3 (13.0%)	20 (12.7%)	1.027	0.28 – 3.774	0.967
EEG abnormalities characterized by an epileptiform wave	17 (73.9%)	82 (52.2%)	2.591	0.971 – 6.919	0.057
Abnormal EEG characterized by a slow wave	8 (34.8%)	39 (24.8%)	1.614	0.636 – 4.095	0.314

The bold in the table indicates statistically significant data.

A good outcome was defined as a seizure-free remission for at least one year. A poor outcome was defined as no seizure-free remission for one year. The percentages sum up by column.

**Table 4:** Multivariate logistic regression analysis for predictors of poor outcome in the newly diagnosed epilepsy

**Predictor**	**Outcome**		**Multivariate analysis**		
	**Poor (N = 23)**	**Good (N = 157)**	**OR**	**95% CI**	***P *value**
Multiple seizure type	10 (43.5%)	27 (17.2%)	3.33	1.29 – 8.60	**0.013**
Changes in seizure type during the treatment	3 (13.0%)	3 (1.9%)	5.88	1.03 – 33.62	**0.047**

The bold in the table indicates statistically significant data.

A good outcome was defined as a seizure-free remission for at least one year. A poor outcome was defined as no seizure-free remission for one year. The percentages sum up by column.

## Discussion

To date, the clinical patterns of epilepsy remain poorly understood, although three different patterns of DRE have been proposed [15,16]: the *de novo* continuous drug resistance, reversal of drug resistance, and progression to drug resistance. If different patterns exist, their recognition would be useful for counseling and planning interventions for patients with epilepsy.

The results of our study showed that 144 patients achieved remission during the follow-up period, and 17 out of 19 patients who entered late remission also remained in terminal remission with no relapse, suggesting that initial failure to enter remission cannot reliably indicate a long-term failure to achieve remission. In agreement with our data, Camfield et al. [17] found that 61% of 345 children responding to the first AED eventually went into remission. Further, 30 out of 72 (42%) children, who failed to respond to the first AED, later achieved remission thus suggesting that initial drug response cannot reliably predict drug resistance. Out of 125 patients entering early remission, 91 patients remained in remission at the end of the follow-up period, suggesting a remitting course. Furthermore, 27.2% of the patients who reached early remission were unable to regain remission after relapse, indicating a progressively worsening course of epilepsy. In 19 out of 180 (10.5%) patients, remission was followed by relapse and return to terminal remission, which suggested a remitting–relapsing pattern of epilepsy. In a recent study from British, Brodie et al. [18] delineated four temporal patterns of outcome in newly diagnosed epilepsy: A) early and sustained seizure freedom; B) delayed but sustained seizure freedom; C) fluctuation between periods of seizure freedom and relapse; and D) seizure freedom never attained. At the end of follow-up, a total of 1,098 patients were included, 749 (68%) patients were seizure-free. Outcome pattern A was observed in 408 (37%), pattern B in 246 (22%), pattern C in 172 (16%), and pattern D in 272 (25%) patients. The results were similar with our study, though the proportions of pattern B and D were slightly higher than ours, which may be explained by the differences in the selection criteria or genetic background.

In our study, 144 newly diagnosed epilepsy patients achieved initial remission with AEDs treatment for at least one year. The majority of relapses occurred within three years after initial one-year remission achievement. As 48 patients were lost during follow-up, which caused the censored data was large and the latter part of the survival plot was not very reliable, we only analyzed the data from the first three years. As illustrated in the Kaplan-Meier survival plot (Figure 2), the relapse rates were 19.5% at 2 years and 31.9% at 3 years during the follow-up. We have noticed that this data is different from another recent study from British. Mohanraj and Brodie [19] have reported that 504 of 780 patients in their study achieved remission for at least one year; only 42 patients relapsed during the follow-up period of 12 years. Also in that report, the Kaplan-Meier plot of time to relapse showed a rate of 10.4% after 8 years of follow-up. The higher rate of relapse in our study may also be related to the differences in the selection criteria or genetic background.

Uncontrolled epilepsy has an adverse impact on quality of life [20,21]. Therefore, identification of the time point when drug resistance occurs may be useful to develop alternative interventions to prevent some forms of epilepsy from becoming drug resistant. We do not know when drug resistance develops in the course of epilepsy, which may lead us to miss the optimal time for intervention [22].

There are three concurrent hypotheses about the evolution of drug resistance. The de novo theory stipulates that in most cases, drug resistance has been fully developed before the first seizure or at least before the start of AED therapy [16]. Findings by Kwan et al. [23] support this hypothesis. Further, our study shows the de novo drug resistance in 23 (12.78%) of 180 patients. Such patients are more likely to have poor response to the first AED prescribed. The second hypothesis indicates that there is progression from remission to drug resistance meaning that some patients develop DRE after initially responding well to the first AED. Supporting this, recent studies [24,25] demonstrate a substantial proportion of epilepsy with the childhood onset that does not become drug resistant for many years after the onset. The third hypothesis is that drug resistance is reversible, i.e., it may remit and reappear during the course of epilepsy or the associated therapy. The reverse process is well known as an intermittent pattern in which periods of remission are followed by periods of uncontrolled seizures. Findings from randomized placebo-controlled add-on trials indicate that a small percentage of patients with previous drug-resistant partial epilepsy responded to the therapy and became seizure-free during trials with new AEDs [26]. This type of pattern also existed in our study. As mentioned above, 27.2% of our patients entering early remission were unable to reach remission again in the subsequent follow-up. Such patients could not be classified as DRE according to the definition proposed by the ILAE in 2009 [14]. In our opinion, this was not appropriate because their prognosis is unfavorable. Therefore, the definition of DRE should be revised, and the observed time should be determined in additional studies.

An important characteristic of DRE is that most patients with intractable epilepsy are resistant to most or all AEDs. Current AEDs do not seem to prevent or reverse drug resistance in most patients [16]. At present, there are nearly 20 AEDs available to clinicians. The number of AEDs that needs to have failed in order to define DRE in a given patient has been debated in the literature [6,7,27,28]. The consensus regarding the number of AED failures seems to be 2 or 3. Kwan et al. [23] found that there was a clear negative association between the number of AED regimens tried and the chances of achieving substantial remission. In their study, the respective chances of achieving remission for one year with the first, second and third AED attempts were 47%, 13% and 1%. The findings of Mohanraj et al. [19] were similar with the results of the aforementioned studies. Our results showed that overall rates of achieving a one year remission with the first, second, third and fourth AED trials were 60.0%, 16.6%, 2.8% and 0.6%, respectively. None of the patients who had experienced failure with four AED regimens became seizure-free.

Due to different study designs and the lack of a standard definition of pharmaco-resistance in the literature, the reported incidence of DRE varies from 7% to 36.7% [23,29-31]. To the best of our knowledge, no data has been reported regarding the incidence of DRE in the Chinese population. Therefore, we set up this investigation. Here we reported that the incidence of DRE in east China newly diagnosed epilepsy patients was approximately 13%. In this study, 20% of the patients failed to achieve remission, including 7% of patients were treated with only one AED throughout the period of follow-up (which did not meet the criteria of DRE). It is noteworthy that the duration of follow-up will critically affect the defined course of epilepsy, especially in terms of relapsing and DRE. Some patients with a relative short period of clinical follow-up in this study may influence the result and the incidence of DRE. For instance, some of the above mentioned 7% of patients may develop to DRE in the future study with longer follow-up time.

It is widely recognized that early identification of patients who are at high risk of developing DRE is important. A number of studies were published about the predictive factors for DRE such as the age of seizure onset, frequent seizures before treatment, seizure type, early mental retardation, brain imaging and EEG abnormalities [5-7,23,29,30,32-36]. The two most prominent risk factors of poor outcome identified in the present study were multiple seizure types and change in seizure type during treatment.

## Conclusions

An array of diverse dynamic changes occurs during the course of epilepsy. Our results show that the prognosis of the majority of patients with newly diagnosed epilepsy is good. The chances of seizure control will decline with subsequent treatment regimens after the failure of the first AED treatment. Multiple seizure types and change in seizure type during treatment will predict the poor control of seizures. Patients with these risk factors should receive formal antiepileptic treatment or alternative treatments such as surgery. However, further studies with large sample size, multi-center and long-term follow-up are required to verify our observations.

## Abbreviations

AED: Antiepileptic drug; DRE: Drug-resistant epilepsy; ILAE: International League Against Epilepsy; EEG: Electroencephalography; MRI: Magnetic resonance imaging; OR: Odds ratio; CI: Confidence interval.

## Competing interests

The authors declare that they have no competing interests.

## Authors’ contributions

YFZ was the principal investigator of the main research project was primarily responsible for the conduct of the study. She developed and managed the study database and drafted the manuscript. NY and LYS were the project coordinators and participated in formulating research questions, checking data for quality control. NY performed the statistical analysis. LYS contributed to image treatment and manuscript revision. QD conceived of the study, participated in its design and coordination and helped to draft the manuscript. All authors read and approved the final manuscript.

## Authors’ information

Department of Neurology, Nanjing Brain Hospital affiliated to Nanjing Medical University, 264 Guangzhou Road, Nanjing, Jiangsu Province, China.

## Pre-publication history

The pre-publication history for this paper can be accessed here:

http://www.biomedcentral.com/1471-2377/13/116/prepub
